# The Role of SWI/SNF Complex in Bladder Cancer

**DOI:** 10.1111/jcmm.70348

**Published:** 2025-01-08

**Authors:** Zixiao Lei, Yanfeng Han, Jiejun Liao, Xiaohong Li, Qisheng Su, Zheng Yang

**Affiliations:** ^1^ Department of Clinical Laboratory The First Affiliated Hospital of Guangxi Medical University Nanning Guangxi Zhuang Autonomous Region China; ^2^ Key Laboratory of Clinical Laboratory Medicine of Guangxi Department of Education Nanning Guangxi Zhuang Autonomous Region China

**Keywords:** bladder cancer, personalised treatment, SWI/SNF complex

## Abstract

Bladder cancer originates from bladder tissues and is the ninth most common type of cancer worldwide. The SWI/SNF (SWItch/sucrose non‐ fermentable) complex plays a crucial role in regulating various biological processes, such as cell cycle control, DNA damage repair and transcription regulation. The purpose of this article is to examine the functional studies of the SWI/SNF complex in bladder cancer, highlighting new pathways for creating personalised treatment approaches for bladder cancer patients with mutations in the SWI/SNF complex. By acquiring a comprehensive understanding of the mechanisms of the SWI/SNF complex in bladder cancer, we can offer more precise and effective solutions to treat this disease.

## 1 Introduction

Globally, bladder cancer ranks as the ninth most prevalent form of cancer, with approximately 614,000 new cases and 220,000 fatalities projected for 2022 [1]. There is a significant gender disparity in the incidence and mortality rates of bladder cancer. Globally, the incidence rate ranks sixth and the mortality rate ninth among males [1]. Smoking is a crucial risk factor for the development of bladder cancer, with approximately 50% of cases attributed to this factor [2]. The development of bladder cancer is linked to the emergence of oncogenic mutations and genetic changes in normal cells [3], and mutations in chromatin regulators (KDM6A, KMT2D, KMD2C, CREBBP and EP300), and AT‐Rich Interactive Domain‐containing protein 1A (ARID1A) are common [2], more than 65% of patients with non‐muscle‐invasive bladder cancer have been found to have inactivation of one or more of these regulators, with ARID1A mutations being more common in T1 stage tumours [4]. Bladder cancer presents a significant menace to human health, and delving into treatment modalities at the molecular level will pave the way for personalised bladder cancer therapy.

The SWI/SNF complex, widely distributed in eukaryotes, comprises a set of chromatin remodelling factors. It modulates chromatin structure by harnessing adenosine triphosphatase (ATPase) to hydrolyze ATP and generate energy, thereby enhancing DNA accessibility for transcription factor binding and gene transcription regulation [5]. The involvement of the SWI/SNF complex in the progression of various cancer types has been extensively documented. Notably, the absence of the BRG1‐ and BRM‐associated factor 47 (BAF47) (it is encoded by SMARCB1) subunit due to deletion mutation has been associated with the onset of childhood rhabdoid tumour, representing a significant milestone in establishing a link between the mammalian SWI/SNF complex and cancer [6]. Mutations in ARID1A have been identified in approximately 50% of ovarian clear cell carcinomas and ovarian endometrioid carcinomas [7]; multi‐genomic sequencing technologies have confirmed that SWI/SNF is the most frequently mutated chromatin remodelling complex in human cancers, with the overall incidence of mutations in its subunits being approximately 20% across various cancers [6]. These studies all indicate a close association between the SWI/SNF complex and various cancers.

Given the implication of SWI/SNF complex subunits in diverse cancer types, it is conceivable that the SWI/SNF complex may exert a substantial influence on bladder cancer. This review presents an outline of the mechanisms by which the SWI/SNF complex functions in relation to bladder cancer.

## 2 The Basic Structure and Function of the SWI/SNF Complex

The regulation of chromatin structure encompasses a diverse array of mechanisms, including histone modifications, DNA modifications and ATP‐dependent chromatin remodelling. ATP‐dependent chromatin remodelling factors can be further categorised into families based on their subunit composition and biochemical activities, including the SWI/SNF, INO80, SWR1, ISWI and NURD/Mi2/CHD complexes [8]. Among these, the SWI/SNF complex is primarily associated with regulating chromatin accessibility [5].

**FIGURE 1:**
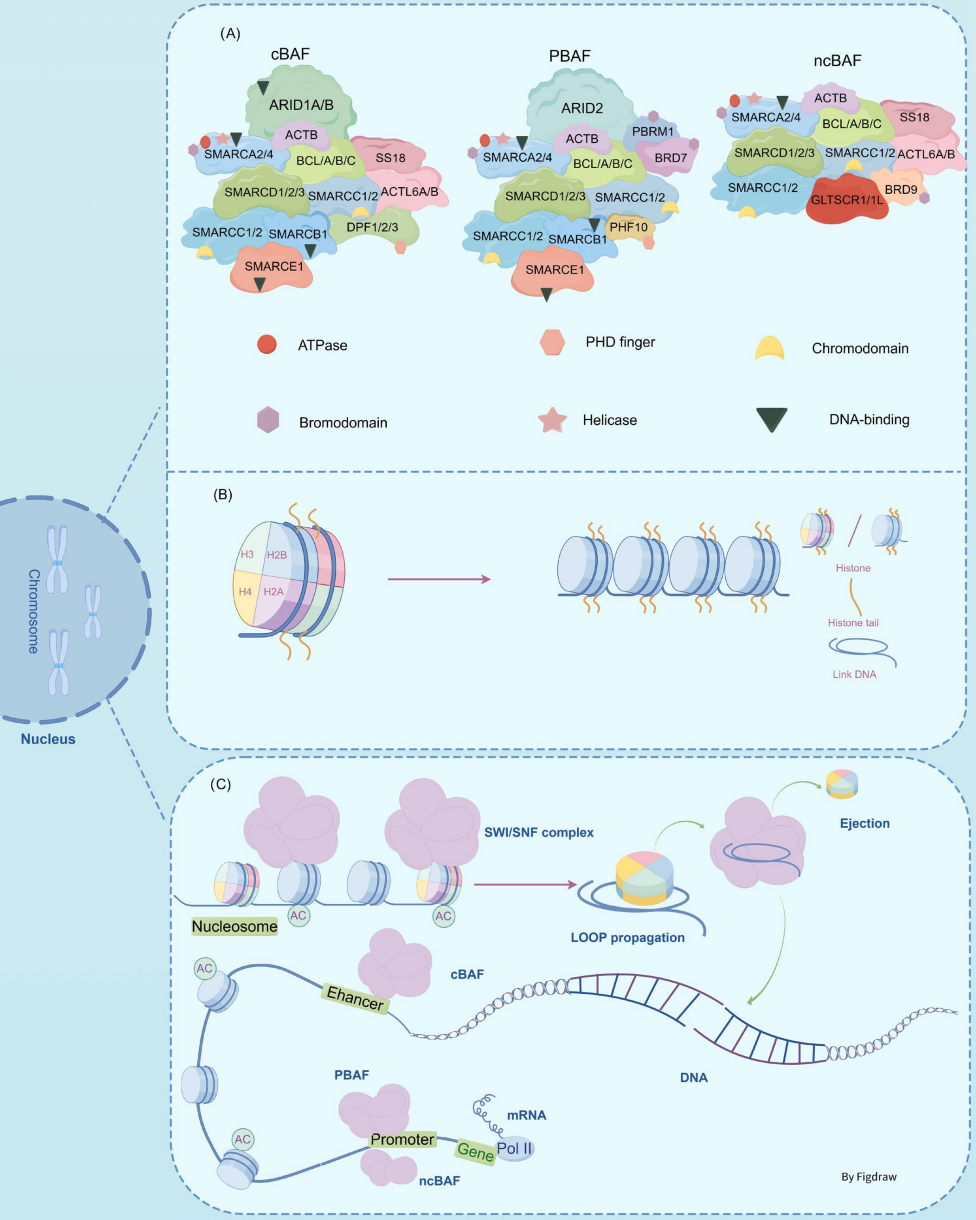
The fundamental architecture and role of the SWI/SNF chromatin remodelling complex. (A)The subunit compositions of different variants of the SWI/SNF complex. (B) The core particle of the nucleosome consists of approximately 147 base pairs of DNA wrapped around a histone octamer core. This histone core is composed of two molecules each of H2A, H2B, H3, and H4, forming a histone octamer. The DNA double helix coils around the histone octamer for about 1.75 turns. (C) The fundamental process of nucleosome mobilisation encompasses: Binding of the SWI/SNF complex to a fixed position on nucleosomal DNA; disruption of histone‐DNA contacts; following ATPase subunit‐mediated DNA translocation and DNA loops formation; the resulting structure can then propagate around the nucleosome, creating accessible sites for DNA‐binding factors.

Two genetic screens in *Saccharomyces cerevisiae* resulted in the identification of genes encoding components of the SWI/SNF complex. The SWI/SNF complex is a multi‐subunit structure (Table 1), consisting of the canonical BAF complex (cBAF) [9] (Figure 1A), the Polybromo‐associated BAF (PBAF) [10] (Figure 1A) and the non‐canonical BAF complex [11] (ncBAF) (Figure 1A) based on its subunit composition. The SWI/SNF complexes each contain either Brahma related gene 1 (BRG1) (it is encoded by SMARCA4) or Brahma related gene product on mating type (BRM) (it is encoded by SMARCA2) as a mutually exclusive ATPase subunit. Additionally, all SWI/SNF complexes include the core subunits BRG1‐ and BRM‐associated factor 155 (BAF155) (it is encoded by SMARCC1), BRG1‐ and BRM‐associated factor 170 (BAF170) (it is encoded by SMARCC2), and BAF47/SNF5 (it is encoded by SMARCB1), along with several variable subunits [12].

**TABLE 1:** The subunits of cBAF, PBAF, ncPBAF.

cBAF	PBAF	ncPBAF	Alisa	Function
**Shared subunits**				
ACTB	ACTB	ACTB		ARP(Actin‐related protein), ATPase activity regulation.
ACTL6A	ACTL6A	ACTL6A	BAF53A	ARP(Actin‐related protein), ATPase activity regulation.
ACTL6B	ACTL6B	ACTL6B	BAF53B	ARP(Actin‐related protein), ATPase activity regulation.
BCL6A	BCL6A	BCL6A		ARP(Actin‐related protein), ATPase activity regulation.
BCL6B	BCL6B	BCL6B		ARP(Actin‐related protein), ATPase activity regulation.
BCL6C	BCL6C	BCL6C		ARP(Actin‐related protein), ATPase activity regulation.
SMARCA2	SMARCA2	SMARCA2	BRM/BAF190B/SNF2L2	It possesses helicase and ATPase activities. It interacts with diverse transcription factors and some DNA assembly proteins, taking part in chromatin structure remodelling and regulating gene expression.
SMARCA4	SMARCA4	SMARCA4	BRG1/BAF190A	Its primary sequence is similar to that of the BRM protein. Its helicase and ATPase activities are capable of regulating the transcription of certain genes through altering chromatin structure.
SS18		SS18	SSXT/SYT	ARP, ATPase
SS18L1		SS18L1	CREXT	ARP, ATPase
SMARCC1	SMARCC1	SMARCC1	BAF155	Its protein contains a leucine zipper motif similar to many transcription factors.
SMARCC2	SMARCC2		BAF170	The BAF170 protein also encompasses a predicted leucine zipper structural domain, which is characteristic of numerous transcription factors.
SMARCD1	SMARCD1	SMARCD1	BAF60A	Core subunit, stabilising the complex structure
SMARCD2	SMARCD2	SMARCD2	BAF60B	Core subunit, stabilising the complex structure
SMARCD3	SMARCD3	SMARCD3	BAF60C	Core subunit, stabilising the complex structure
SMARCB1	SMARCB1		BAF47/INI1/SNF5	DNA‐binding domain
SMARCE1	SMARCE1		BAF57	DNA‐binding domain
**Specific subunits**				
ARID1A			BAF250A/OSA1	First, it possesses a DNA‐binding structural domain that specifically binds to AT‐rich DNA sequences. Secondly, the C‐terminus of this protein stimulates glucocorticoid receptor‐dependent transcriptional activation.
ARID1B			BAF250B/OSA2	It encodes a protein of the AT‐rich DNA interaction domain and may exert a role in cell cycle activation.
DPF1			BAF45B /NEUD4	With a zinc finger structure, it may be related to binding with DNA.
DPF2			BAF45D/REQ/ UBID4	With a zinc finger structure, it may be related to binding with DNA.
DPF3			BAF45C/CERD4	With a zinc finger structure, it may be related to binding with DNA.
	ARID2		BAF200	ARID2 possesses a conserved N‐terminal ARID region and two conserved C‐terminal C2H2 Zn‐finger motifs that either bind directly to DNA or interact with other proteins.
	PHF10		BAF45A	With a zinc finger structure, it may be related to binding with DNA.
	PBRM1		BAF200	Other subunit structural domains collaborate with them to sustain the structure of the SWI/SNF complex. From a functional perspective, the bromodomain is capable of specifically recognising and binding acetylated lysine residues on the tails of histone proteins.
	BRD7			
		BRD9		
		BRCIA	GLTSCR1	GLTSCR1 and GLTSCR1L is considered as glioma tumour suppressor candidate
		BRCIAL	GLTSCR1L	GLTSCR1 and GLTSCR1L is considered as glioma tumour suppressor candidate

*Note:* The SWI/SNF chromatin remodelling complex contains multiple protein subunits, each with different functions. One component of the SWI/SNF complex is well‐known by several alternative names, which are integrated here to enhance its accessibility and comprehension.

Nucleosomes consist of 146 bp of DNA wrapped around an octamer of histone proteins, functioning as the primary structural component of eukaryotic chromatin [13] (Figure 1B). The SWI/SNF complex possesses the capacity to alter nucleosome structure through its ATPase activity, harnessing energy from ATP hydrolysis to mobilise nucleosomes by sliding and catalysing the ejection and insertion of histone protein octamers, this process reduces chromatin density, exposing DNA to facilitate gene expression [5]. The fundamental process of nucleosome mobilisation is shown in Figure 1C.

The ATPase activity of the SWI/SNF complex is believed to govern the assembly of its subunits at genomic loci, each subunit exhibits distinct chromatin localization and the versatility of functions (Figure 1C) [11]. For instance, the SWI/SNF complex is typically localised in regions marked by H3K27 acetylation (H3K27ac), where it collaborates with transcription factors to facilitate a more permissive chromatin conformation [14]; the activity of cBAF (subtypes of the SWI/SNF complex) is expected to be most prominent in enhancer regions, while PBAF (subtypes of the SWI/SNF complex) and ncBAF (subtypes of the SWI/SNF complex), although they also associate with certain enhancers, are reported to have a greater tendency to be enriched in the promoter regions of genes [13]. While commonly linked with gene activation processes, SWI/SNF complex can also direct nucleosome positioning towards binding transcriptional repressor or establishing chromatin with repressive features [11]. In essence, these findings elucidate distinctive patterns of chromatin localisation exhibited by individual subunits within the SWI/SNF complex.

## 3 The Role of SWI/SNF Complex Subunits in Bladder Cancer

### 3.1 SWI/SNF Complex Regulates Tumour Proliferation, Invasion, and Metastasis

**FIGURE 2:**
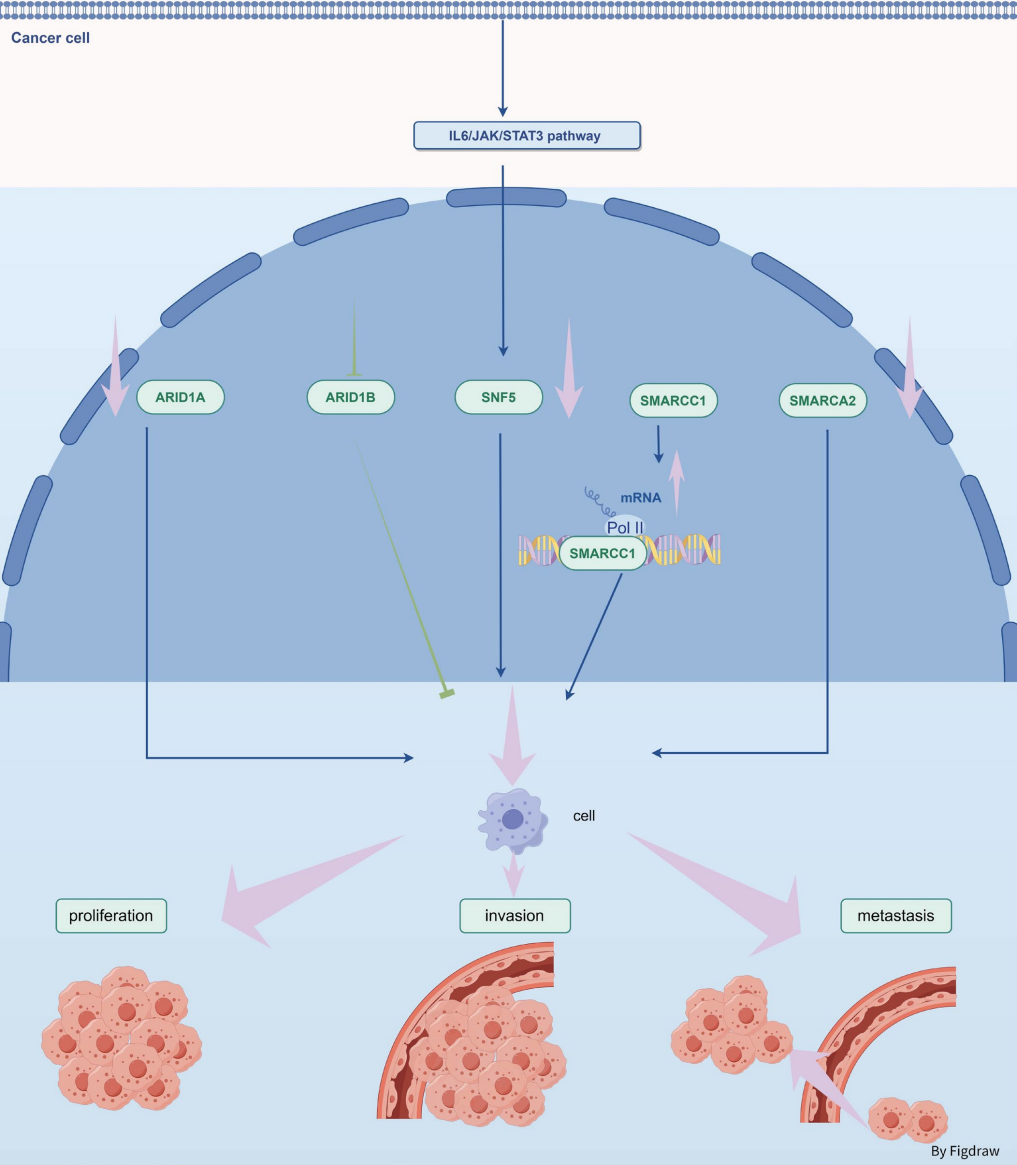
The SWI/SNF complex plays a critical role in modulating tumour proliferation, invasion, and metastasis. The expression loss of ARID1A, SMARCA2, and SMARCB1 in bladder cancer is associated with tumour grade and stage. Inhibition of ARID1B expression significantly reduces the proliferation, migration, and invasion capabilities of bladder cancer cells. Elevated mRNA and protein expression levels of SMARCC1 are associated with higher T stages and poorer prognosis in bladder cancer patients.

Studies have suggested a strong correlation between the development of bladder cancer and various subunits within the SWI/SNF complex. (Figure 2). In the BRG1/BRM‐associated factors, ARID1A and AT‐rich interactive domain‐containing protein 1B (ARID1B), which contain abundant AT interaction domains, exist as mutually exclusive isoforms [7]. ARID1A has been demonstrated to function as a critical tumour suppressor [15], and studies have suggested its role in repressing potentially carcinogenic gene expression in bladder epithelial cells by maintaining a balance between transcription and translation [16]. The occurrence rate of ARID1A gene mutation is increased in squamous bladder cancer (Sq‐BLCA) (15%) and correlates with the absence of ARID1A protein [17]. In the context of bladder cancer, the lack of ARID1A protein is associated with tumour grade and stage [18, 19, 20]. As an alternative subunit to ARID1A, ARID1B shares high homology with it but may play a distinct role in tumorigenesis, it is possible that ARID1A and ARID1B have opposing functions in tumour development, with ARID1A crucially inhibiting cell proliferation while ARID1B appears to promote cell proliferation and maintain stem cell characteristics [21]. Inhibiting the expression of ARID1B significantly reduces the proliferation, migration and invasive ability of bladder cancer cells, suggesting that it could serve as a potential prognostic biomarker for urothelial carcinoma of the bladder and aid in patient selection for adjuvant chemotherapy [22].

Study have demonstrated that absence of SMARCB1 (encoding SNF5/BAF47 protein) leads to enhanced chromatin accessibility at the STAT3 promoter, which in turn triggers activation of the IL6/JAK/STAT3 pathway to facilitate the growth and spread of bladder cancer [23]. The reduced expression of SMARCA2 (encoding BRM protein) has been linked to the metastasis of muscle‐invasive bladder cancer (MIBC) and decreased rates of patient survival [24]. The levels of SMARCC1 (encoding BAF155 protein) mRNA and protein expression are increased in bladder cancer, and this heightened expression is strongly linked to advanced T stage and unfavourable prognosis in patients with bladder cancer [25].

Frequent mutations or downregulation of SWI/SNF complex subunits occur in bladder cancer, closely correlating with invasiveness, malignancy and poor prognosis. These findings underscore the potential significance of targeted therapies directed at the SWI/SNF complex for treating bladder cancer.

### 3.2 SWI/SNF Complex Regulates the Cell Cycle

**FIGURE 3:**
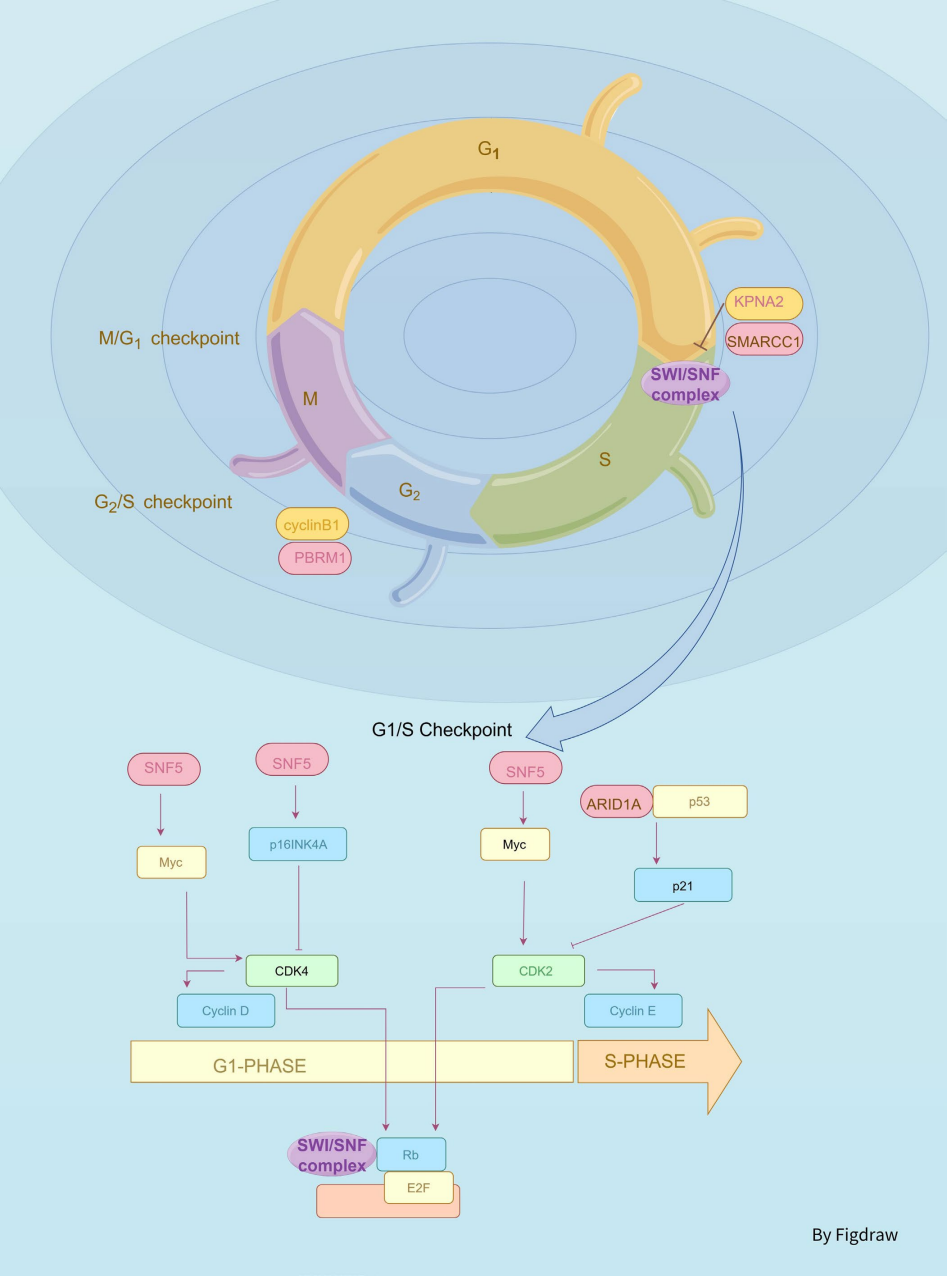
The SWI/SNF complex plays a critical role in the regulation of the cell cycle. The SWI/SNF complex interacts with cell cycle‐related regulatory factors, with the complex primarily functioning during the G1/S phase. At the end of G1 phase, CDK4/6 binds to cyclin D to form a complex, promoting the phosphorylation of the Rb protein. Subsequently, CDK2 binds to cyclin E, further phosphorylating Rb and releasing the transcription factor E2F, thereby promoting the transcription of S phase genes. MYC promotes the transition of cells from G1 to S phase by upregulating the expression of cyclins and cyclin‐dependent kinases. p21 can prevent cells from entering S phase by inhibiting the activity of CDK complexes. The SWI/SNF complex can bind to RB and regulate the activity of E2F; SNF5 can inhibit MYC, and its inactivation can lead to the downregulation of p16INK4A; the subunits of SWI/SNF can also regulate the p53/p21 pathway, for example, ARID1A acts in concert with p53 to induce tumorigenesis, and it can also affect p21. PBRM1 can inhibit cyclin B1; SMARCC1 enters the nucleus through KPNA2, affecting the cell cycle.

The inactivation of SWI/SNF has distinct effects on regulation of the cell cycle compared to G2/M phase arrest caused by DNA damage; it plays a pivotal role in G1 phase and G1‐S transition [24](Figure 3).

The SWI/SNF complex binds to retinoblastoma protein (RB) and influences the repression of RB target genes [26]. This pathway regulates the transition from the G1 phase to the S phase of the cell cycle and is also involved in DNA repair, DNA synthesis and cell division [27]. p16INK4A, an inhibitor of cyclin‐dependent kinase (CDK), modulates the RB tumour suppressor pathway by restraining cyclin D1‐CDK4 mediated RB phosphorylation, thereby exerting pivotal influence on cell cycle regulation and differentiation [28], its downregulation subsequent to SNF5 (it is encoded by the SMARCB1) inactivation is associated with tumorigenesis [28]. RASSF6, a tumour suppressor protein that belongs to the RASSF family, is epigenetically silenced in numerous human cancers. Its low expression is associated with an unfavourable prognosis. The overexpression of RASSF6 can induce cell apoptosis and cell cycle arrest. Moreover, DNA damage triggers the activation of cyclin‐dependent kinase 9 (CDK9) to phosphorylate BAF53 (It is encoded by the ACTL6A or ACTL6B), causing RASSF6 to remain in the cell nucleus and promoting the formation of the BAF53‐BAF60A‐p53 complex, thereby activating the transcription of p53 target genes (such as BAX, BTG2 and CDKN1A genes) and consequently influencing the regulation of the cell cycle [29]. BAF57 (it is encoded by the SMARCE1), BAF60A (It is encoded by SMARCD1) and SNF5 can bind to p53 and activate the p53/p21 and p16/pRB ageing pathways to promote cell cycle arrest and cell ageing [30]. Dasatinib selectively exerts cytotoxicity on ARID1A mutant ovarian clear cell carcinoma (OCCC) cells. Following dasatinib treatment, ARID1A mutant OCCC cells exhibit G1 phase arrest and increased apoptosis. Dasatinib may exert inhibitory effects on ARID1A‐deficient tumour cells by regulating the cell cycle regulatory network associated with cyclin—dependent kinase inhibitor 1A (CDKN1A) and RB1 [31]. ARID1A interacts with Rb to prevent CDK‐mediated Rb phosphorylation, inhibit E2F transcription factor 1 (E2F1) activity, and subsequently inhibit c‐Myc transcription, revealing a new mechanism of ARID1A in squamous cell carcinoma (SCC). ARID1A deficiency leads to increased cancer stemness through epithelial‐mesenchymal transition (EMT), causing SCC chemotherapy resistance. Cyclin‐dependent kinase inhibitor (CDKi) can restore chemosensitivity in resistant cells by inhibiting the pRb/E2F1/c‐Myc pathway, providing a new strategy for treating SCC patients with low ARID1A expression [32]. From the above research, we can see that the various subunits of the SWI/SNF complex plays an important role in regulating the cell cycle, especially in the p53, RB pathway, but these studies are relatively scarce in bladder cancer.

In bladder cancer, studies have demonstrated a high prevalence of RB1 gene mutations [33]. This discovery implies that the interplay between the SWI/SNF complex and the RB in bladder cancer may encompass their roles in governing the expression of genes and cell cycle advancement. In the setting of bladder cancer, the absence of ARID1A could potentially impact the regulation of the cell cycle‐associated gene p21, leading to potential alterations in the growth and propagation of tumour cells [18]. The sole deletion of ARID1A does not induce bladder tumour development in mice [34], and previous studies have suggested that the absence of ARID1A function alone may not be adequate to initiate tumorigenesis, and that the concurrent loss of TP53 gene function is a pivotal driver for carcinogenesis in urothelial cells [18], indicating an important connection between the cell cycle‐related genes and the SWI/SNF complex. Additionally, SNF5/BAF47 (it is encoded by the SMARCB1) has traditionally been regarded as a tumour suppressor protein capable of exerting tumour suppression by inhibiting MYC activity [35]. As yet, our understanding regarding the impact and specific mechanisms underlying SNF5/BAF47 (it is encoded by the SMARCB1) in bladder cancer remains incomplete. PBRM1 (encoding BAF180 protein) is found to be mutated in 2%–10% of bladder tumours, and it exerts a suppressive effect on bladder cancer by inhibiting the activity of cyclin B [36]. SMARCC1 (encoding BAF155 protein) translocates into the nucleus via KPNA2 and functions as an oncogenic gene, downregulation of SMARCC1 (Encoding BAF155 protein) expression results in G1/S phase arrest and an increase in apoptotic cells in bladder cancer [25]. In conclusion, SWI/SNF regulation of the cell cycle may have a significant impact on the development of bladder cancer.

### 3.3 The SWI/SNF Complex Regulates DNA Repair

Double‐strand breaks (DSBs) are regarded as the most genetically toxic form of DNA damage [37]. To recruit and activate the key factors involved in the repair process, the DNA damage response signalling pathway is activated. DSBs are primarily repaired through two pathways, namely, non‐homologous end joining (NHEJ) and homologous recombination (HR) [15]. Chromatin remodelling factors, such as the SWI/SNF complex, can mediate histone modification and nucleosome disassembly, thereby relaxing chromatin and facilitating DNA repair [38].

The BRG1 (it is encoded by the SMARCA4) subunit has been extensively studied in the context of DNA damage repair; however, the influence of BRG1 on DSB repair pathways and the mechanism through which it promotes repair vary among different models. Some studies support that BRG1 (It is encoded by the SMARCA4) promotes HR repair by interacting with RAD52 to replace Replication Protein A (RPA) with RAD51 [39]; others suggest that BRG1 is recruited to DSBs through interaction with the TopBP1‐E2F1‐RB complex [26]. Furthermore, the study ascertained that E1A‐binding Protein p400‐associated Factor 1 (E4F1) is promptly recruited to DNA damage sites by Poly (ADP‐ribose) polymerase‐1 (PARP‐1) activity through the observation of endogenous E4F1 protein and E4F1 protein tagged with green fluorescent protein (GFP), and that E4F1 facilitates DNA end resection and HR via its interaction with BRG1. This is pivotal for attaining transcriptional silencing at the site of DNA double‐strand break; in addition, the E2F1‐pRB complex also participates in the recruitment of BRG1 to the site of double‐strand break, and this study also affirmed that both pRB‐E2F1 and E4F1 might be involved in the recruitment of BRG1 [40]. These studies reveal a close association between BRG1 and DNA damage repair. Radiofrequency interstitial tumour ablation (RITA) may trigger DNA damage signalling, inducing the activation of the ATM/CHK2 pathway, which subsequently phosphorylates p53. The phosphorylation state of p53 changes after that, leading to changes in its conformation or activity. This indirectly interferes with the normal interaction between p53 and mouse double minute 2 (MDM2), resulting in an increase in p53 protein levels and affecting cell growth, apoptosis. Meanwhile, ARID1A participates in DNA damage repair, and its absence makes it easier for RITA‐induced DNA damage to accumulate, ultimately leading to cell death [41]. After DNA damage, ARID1A accumulates at DSBs and regulates chromatin looping by recruiting RAD21 and CCCTC—binding factor (CTCF) to DSBs. To avoid conflicts between repair and transcription, ARID1A also promotes transcriptional silencing at DSBs in transcriptionally active chromatin by recruiting Histone Deacetylase 1 (HDAC1) and remodelling and spacing factor 1 (RSF1) [15]. A study conducted using mouse xenograft experiments demonstrated that cells lacking ARID1A are sensitive to poly (ADP ‐ribose) polymerase inhibitor (PARPi), and when PARPi is combined with low‐dose ionising radiation, the toxicity of PARPi can be enhanced [42]. Glucocorticoids are widely used clinically due to their potent anti‐inflammatory properties and are also widely employed in cancer treatment as they can induce apoptosis and promote cell cycle arrest. ARID1A can influence the glucocorticoid pathway to regulate the cell cycle and DNA damage. Knockdown of ARID1A disrupts chromatin‐associated protein complexes of various DNA damage repair proteins (such as P53BP1, PARP1 and DDB1) and histone acetyltransferase KAT7, affecting the interaction of glucocorticoid receptor (GR) with these proteins, thereby influencing DNA repair and cell cycle regulation. ARID1A knockdown also affects P53‐related genes (KLF4, TSC22D1, BTG1, FOS and S100A10) and prolongs the G1 phase cell cycle arrest regulated by dexamethasone [43]. In ARID1B‐deficient cells, the levels of phosphorylated CHK1, CHK2, ATM and γH2AX are elevated, indicating significant DNA damage [44]. ARID1B and ARID1A are mutually exclusive and exist in the cBAF complex (subtypes of the SWI/SNF complex). Its inactivation in certain cells leads to NHEJ defects, and co‐inactivation with ARID1A causes the inactivation of the cBAF complex (subtypes of the SWI/SNF complex). Targeting ARID1B in ARID1A‐mutated cells increases their sensitivity to infrared radiation (IR); ARID1B knockdown selectively increases the radiation sensitivity of ARID1A‐mutated colorectal cancer (CRC) cells, which is related to the suppression of RAD51 focus formation, and is not dependent on the antiproliferative effect, suggesting that ARID1B may be a potential therapeutic target to enhance the radiotherapy sensitivity of ARID1A‐deficient tumours, and its function in DNA repair still requires further investigation [45]. A recent study investigated the role of bromodomain‐containing protein 7 (BRD7) in transcriptional repression that occurs near double‐strand breaks. After DNA damage, ATM and ATR phosphorylate BRD7, and these phosphorylation events are crucial for the interactions between various repair proteins and BRD7 [46].

Radiation therapy and chemotherapy eliminate cancer cells by inducing DNA damage (including DSB), and mutations in the SWI/SNF complex result in DNA repair deficiencies, which can serve as a therapeutic target [6]. Research has demonstrated that cisplatin‐induced activation of the NFkB signalling pathway can enhance the transcription of N‐acetyltransferase 10 (NAT10), which regulates the expression of AHNAK and other genes to facilitate DNA damage repair in bladder cancer cells, thereby augmenting cisplatin resistance. This indicates a close association between DNA damage repair and bladder cancer treatment [47]. Nevertheless, no research has verified the mechanism of the SWI/SNF complex and DNA damage in bladder cancer. However, in bladder cancer, mutations occur in multiple subunits of the SWI/SNF chromatin complex [2, 48], it has disclosed a potential connection between the SWI/SNF chromatin complex and DNA damage, which enables us to formulate relevant treatment notions based on the mechanisms of DNA damage. In bladder cancer, a disorder exists in the subunits of various SWI/SNF chromatin complexes [24]. The inactivation of BRG1 renders cells susceptible to DNA damage, and the development of ATPase inhibitors might be beneficial for cancer treatment [26, 39, 49]. ARID1A mutations are prevalent in various cancers, and the mutated cells are sensitive to PARPi inhibitors [50]. ARID1B, as a homologous subunit of ARID1A, can potentially serve as a target for ARID1A‐mutated bladder cancer [45]. The inactivation of BRD7 increases the sensitivity of cells to Camptothecin (CPT) and PARPi. The inactivation of BRD7 leads to an increased sensitivity of cells to CPT, ionising radiation (IR), and PARPi. Their combined use leads to accelerated cell damage and also provides novel ideas for treatment approaches [46]. The role of the SWI/SNF complex in DSB repair is gradually becoming clear, but there remain numerous issues that require further investigation, such as the specific functions of each subunit in DSB repair and the interaction between different ATPases during DNA end resection. In the future, superior tools and methods need to be developed to conduct in‐depth studies on its function to advance the treatment of SWI/SNF complex mutated cancers.

### 3.4 SWI/SNF Complex Governs the Regulation of Immune Checkpoints

Programmed death protein 1 (PD‐1) is an immune checkpoint protein expressed by T cells during initial antigen activation, under physiological conditions, the interaction between PD‐1/programmed cell death ligand 1 (PD‐L1) and PD‐1/programmed death ligand 2 (PD‐L2) contributes to maintaining immune system tolerance [51]. In the tumour microenvironment, tumour cells can exploit the PD‐1 pathway to evade immune system surveillance and attack. Various immune checkpoint therapies targeting PD‐1 or PD‐L1 have been approved as first‐ or second‐line treatments for metastatic urothelial carcinoma, with a response rate of approximately 30% [52].

Currently, numerous studies have attested to a close association between the SWI/SNF chromatin remodelling complex and immunotherapy. In lung cancer cells where ARID1B is knocked down, the activation of the cyclic GMP‐AMP synthase‐stimulator of interferon genes (cGAS‐STING) pathway is augmented, and this activation improves the efficacy of immunotherapy, the high expression of ARID1B is correlated with immune suppression, low immune scores, decreased immune cell infiltration, and negative correlations with various immune‐related cell types and functions. These results imply that ARID1B might play a crucial role in regulating the immune environment of non‐small cell lung cancer [44]. Loss of ARID1A leads to the accumulation of R‐loops, which generates cytoplasmic DNA that activates the STING‐I type interferon signalling pathway, inducing gene expression that promote anti‐tumour immunity, providing a theoretical basis for improving immune checkpoint blockade (ICB) therapy [53]. Radiation therapy is commonly used in clinical practice to treat certain types of cancer, such as breast cancer. Some studies have used IR to treat ARID1A‐deficient breast cancer cell lines, inducing DNA damage. After IR treatment, the expression of genes Interferon‐α (INF‐α), Interleukin‐6 (IL‐6), Chemokine C‐X‐C motif Ligand 9 (CXCL9), and Chemokine C‐X‐C motif Ligand 10 (CXCL10) increased. Furthermore, the study showed that low ARID1A expression breast cancer patients who received radiation therapy (RT) had higher expression of IL‐6 compared to patients with high ARID1A expression. Low ARID1A expression cancer patients may benefit from a combination of radiation therapy and DNA damage repair inhibitors to increase DNA damage load and/or immunotherapy to further enhance the function and infiltration of immune cells. This treatment combination approach has great potential and needs to be further validated using in vivo models [15].

Bladder cancer samples of the squamous cell type show a lack of ARID1A protein and elevated levels of PD‐L1 expression [17], indicating potential benefits from immune checkpoint inhibitor therapy for these patients. In metastatic urothelial carcinoma, mutations in the ARID1A gene and increased expression of C‐X‐C motif chemokine ligand 13 (CXCL13) in cancer tissue improve sensitivity to immune checkpoint therapy; both can serve as biomarkers predicting efficacy of such therapy [54]. Immune checkpoints could represent an effective treatment option for bladder cancer driven by the SWI/SNF complex. Although there have been limited direct studies targeting this specific aspect in bladder cancer, research in other cancer types suggests that alterations in the SWI/SNF complex can affect the tumour immune microenvironment. In the future, we can deepen our research on this aspect of bladder cancer to provide more possibilities for its treatment.

### 3.5 SWI/SNF Complex Affects the Mechanism of Resistance to Drugs

The advent of acquired resistance constrains the long‐term efficacy of targeted therapies in cancer, and epigenetic processes are capable of mediating resistance to targeted therapies, presenting new therapeutic targets, particularly in tumours lacking explicit genetic resistance mechanisms [55]. Nevertheless, the mechanisms by which epigenetic processes contribute to resistance remain incompletely understood. The mammalian SWI/SNF complex regulates chromatin structure by altering DNA‐nucleosome contact and chromatin accessibility, which may be associated with the development of cancer drug resistance.

Osimertinib, a tyrosine kinase inhibitor (TKI) employed for the treatment of EGFR mutant lung adenocarcinoma, has restricted long‐term efficacy due to acquired resistance to TKI, which is frequently caused by non‐genetic mechanisms. Sensitive and resistant EGFR mutant cells and patients are originated from chromatin accessibility and gene regulatory characteristics. Disrupting the genetic and pharmacological level of SMARCA4/SMARCA2 (Encoding BRG1/BRM protein), which possesses ATPase activity, can render a portion of the resistant model sensitive once again by inhibiting the SWI/SNF‐mediated regulation of cell proliferation, epithelial‐mesenchymal transition, epithelial cell differentiation, and Nuclear factor erythroid 2‐related factor 2 (NRF2) signal transduction, thereby restoring sensitivity to osimertinib. Novel SWI/SNF ATPase inhibitors can collaborate with osimertinib to reverse the resistance in some cell lines, reshape chromatin accessibility and gene expression profiles, and downregulate NRF2 signalling pathway‐related genes, suggesting that it is an effective strategy to overcome resistance [56]. The deficiency of H3K36 methyltransferase NSD1 would render cells resistant to the inhibition of EZH2. The study have demonstrated that NSD1 counteracts Polycomb‐repressive complex 2 (PRC2) through collaboration with SWI/SNF complex, and the study have identified the co‐occurrence of NSD1 inactivation in SWI/SNF‐deficient cancers, which indicates its relevance in vivo [57]. Reduced levels of SNF5/BAF47 (it is encoded by the SAMRCB1) expression could potentially lead to heightened insusceptibility to primary medications like cisplatin and gemcitabine in bladder cancer cells [58]. Phosphoproteomic analysis reveals phosphorylation state of EGFR in cells with SNF5 deletion; inhibiting EGFR in SNF5‐deficient cells can lead to improved therapeutic effects [59]. Bladder tumour cells with the presence of reduced SNF5 expression show increased sensitivity to gefitinib, FDA approved EGFR‐targeted drug for lung cancer treatment [58], EGFR‐targeted drugs may be an effective approach for treating SNF5‐deficient bladder cancer associated with drug resistance. Additionally, an inhibitor of enhancer of zeste homologue 2 (EZH2) can enhance sensitivity of SNF5‐deficient cells to cisplatin, a novel treatment strategy based on SNF5 expression involving EGFR‐targeted chemotherapy or cisplatin combined with EZH2 inhibitor is proposed for bladder cancer management [58].

### 3.6 SWI/SNF Complex Regulates Gene Expression and Repression

Research has demonstrated that the SWI/SNF complex and the Polycomb protein family engage in a mutually antagonistic relationship to regulate gene expression, shedding light on the precise control of gene expression by the SWI/SNF complex [28]. The upregulation of EZH2 is observed in various aggressive cancers, designating it as a driver oncogene for tumour growth [77]. EZH2 functions as an active constituent of the PRC2 and is responsible for histone H3 lysine 27 trimethylation (H3K27me3), leading to transcriptional silencing of numerous genes, including certain tumour suppressor genes [5]. ARID1A‐mutated bladder cancer cells exhibit sensitivity to PI3K inhibitors; thus combining EZH2 inhibitors with PI3K inhibitors can yield a synergistic anti‐tumour effect, consequently, ARID1A mutated bladder cancer may be treated with EZH2 and/or PI3K inhibitors [60]. The dysregulation in the antagonistic interplay between SNF5 (It is encoded by the SAMRCB1) and EZH2 is pivotal in the pathogenesis of tumours propelled by SNF5 inactivation in vivo, the ablation of SNF5 results in heightened expression of EZH2, leading to widespread trimethylation of H3K27, which subsequently represses Polycomb target genes and ultimately contributes to tumorigenesis [61], in specimens from bladder cancer patients, there is a significant downregulation of SNF5 protein expression [24], indicating that SNF5 may impact bladder cancer progression through related pathways. Therefore, targeted EZH2 for the modulation of gene expression could offer therapeutic benefits in the management of bladder cancer characterised by SWI/SNF mutations.

### 3.7 The SWI/SNF Complex Exhibits a Phenomenon Known as ‘Synthetic Lethality’

‘Synthetic lethality’ is a genetic concept that refers to the situation where simultaneous inactivation of two or more genes in a cell results in cell death, while the individual inactivation of any one gene does not lead to cell death [62]. ARID1A is widely recognised as a synthetic lethal gene and shares homology with ARID1B [63]. This discovery presents potential targets for cancer therapy. However, studies have also indicated that double deletion of ARID1A and ARID1B can trigger rapid carcinogenesis in the liver and skin of mice, suggesting caution should be exercised when employing homologous therapy for treating cancers with mutated ARID1 [64]. ARID1A is frequently mutated in various cancers; however, the functional relationship and synthetic lethality mechanism between ARID1A and its homologue ARID1B remain unclear. Our research indicates that ARID1A plays a predominant role in maintaining chromatin accessibility at enhancer sites, while the function of ARID1B is only evident in the context of ARID1A mutation. The ARID1A/1B‐dependent accessible sites are primarily located in enhancer regions, and the accessibility of these sites is associated with the binding of AP‐1 transcription factors. The depletion of ARID1A and ARID1B leads to a reduction in the accessibility of AP‐1 binding sites, a decrease in the distance between nucleosomes, and affects the binding of AP‐1 transcription factors, thereby influencing the expression of multiple cancer‐related genes, including MET [65]. ARID1A mutations can induce cellular damage, and in cells where ARID1A has undergone mutation, targeting ARID1B would enhance the sensitivity of the cells to ionising radiation. When ARID1B is knocked down, a selective increase in the radiation sensitivity of ARID1A mutant CRC cells is witnessed, which is associated with the suppression of RAD51 focus formation and the impairment of Homologous Recombination, suggesting that ARID1B may potentially serve as a therapeutic target for enhancing the radiation sensitivity of ARID1A‐deficient tumours [45].

BRG1 (it is encoded by the SAMRCA4) and BRM (it is encoded by the SAMRCA2) are closely related multi‐domain proteins, both containing a highly conserved ATPase domain that is sensitive to DNA [66], playing a crucial role in chromatin remodelling. Mutations in the BRG1 gene lead to increased reliance on the ATPase activity of BRM for cancer cell growth due to its partial compensation for the functional deficiency of BRG1, this discovery unveils a synthetic lethal relationship between BRM and BRG1, positioning BRM as an attractive anti‐cancer target [67]. Reports have emerged regarding the discovery of a highly potent and selective proteolysis—targeting chimaera (PROTAC) A947, which enables selective degradation of SMARCA2 (encoding BRM protein) and shows effective in vitro growth inhibition and in vivo therapeutic efficacy compared to wild‐type models in SMARCA4 (encoding BRG1 protein) mutant models. This study emphasises the development of PROTACs that selectively and effectively target SMARCA2 in vivo through the conversion of non‐selective SMARCA2/4‐binding ligands, thereby potentially offering a new therapeutic opportunity for patients with tumours harbouring SMARCA4 mutations [68].

In the clinical application of bladder cancer treatment, the identification of a synthetic lethal gene that interacts with the SWI/SNF complex gene may facilitate targeted inhibition of its function using pharmaceutical agents or other modalities. This approach holds promise as an integral component of future personalised cancer therapy within the realm of precision medicine.

### 3.8 The SWI/SNF Complex Regulates Metabolic Processes

The SWI/SNF chromatin remodelling complex is involved in sucrose metabolism in yeast and nutrient sensing and metabolic regulation in mammals [24]. The energy metabolism of cancer cells tends to produce energy through glycolysis (rather than through aerobic energy production via oxidative phosphorylation), which is known as the Warburg effect. This metabolic reprogramming enables cancer cells to produce high levels of energy even in anoxic or hypoxic conditions. The metabolic switch of glucose transporter protein 1 (GLUT1) significantly increases the uptake of glucose by cancer cells [7]. This might imply that the SWI/SNF complex could influence the occurrence and development of cancer by regulating the relevant metabolic pathways.

In lung cancer cells, hypoxia‐inducible factor 1 (HIF1A) is capable of upregulating the expression of GLUT1 or enhancing its activity, thereby facilitating the entry of more glucose into the cells. Glucose serves as the substrate for glycolysis, and the increased glucose influx into the cells supplies ample raw materials for glycolysis, consequently promoting the continuous progression of glycolysis to fulfil the energy requirements of tumour cells [69]. Research findings have indicated that the deficiency of ARID1A strengthens the binding of HIF1A to the promoter region of glycolytic regulatory factor genes. Under such circumstances, it further stimulates the expression of glycolytic regulatory factor genes and thereby augments the generation of glycolytic energy [70]. In bladder cancer, the level of SMARCA2 (encoding BRM protein) is significantly lower than that in normal tissues, and the low expression of SMARCA2 is associated with a poor prognosis in patients. The expression of SMARCA2 is closely related to the expressions of pyruvate kinase M2 (PKM2) and protein kinase AMP‐activated alpha 1 catalytic subunit (PRKAA1) genes as well as metastasis, which suggests that metabolic alterations exist in bladder cancer. Additionally, the expression of SMARCA2 influences the expression of Fructose‐1,6‐bisphosphatase 1 (FBP1), and the low expression of FBP1 is related to the clinical stage of bladder cancer, more frequent recurrence, and metastasis. Therefore, the expression of FBP1 can serve as a prognostic marker for bladder cancer patients [24]. The chromatin remodelling factor BAF60s (it is encoded by the SMARCD) serves as a crucial regulator of energy metabolism, and its dysregulation exerts a key role in the pathogenesis of metabolic syndrome [71]. There have been studies suggesting that BAF60A (it is encoded by the SMARCD1) also plays a role in regulating metabolism in bladder cancer. To explore the molecular mechanism through which the tumour suppressor miR‐99a‐5p regulates the chemoresistance of bladder cancer, a study constructed resistant bladder cancer cells to gemcitabine (GEM—R BC) cells that were resistant to gemcitabine. miR‐99a‐5p functioned as a tumour suppressor in GEM—R BC cells and enhanced their sensitivity to gemcitabine. Further exploration revealed that miR‐99a‐5p directly bound to a specific sequence in the 3'UTR of the SMARCD1 (Encoding BAF60A protein) mRNA. The overexpression of miR‐99a‐5p and the downregulation of SMARCD1 in bladder cancer cells could effectively inhibit the formation and growth of bladder cancer in vivo. The downregulation of SMARCD1 induced the activity of senescence‐associated β‐galactosidase (SA‐β‐gal) and upregulated the expression of p21 waf1/cip1 in protein immunoblotting. Similarly, transfection with miR‐99a‐5p demonstrated these changes, suggesting that the low expression of SMARCD1 and the overexpression of miR‐99a‐5p might be involved in inducing cell senescence, thereby influencing cell proliferation, migration, invasion, and sensitivity to gemcitabine [72].

The metabolic regulation mediated by SWI/SNF holds significant relevance in multiple aspects of the development of bladder cancer, such as cell proliferation, adaptation to the microenvironment, and others. A more profound comprehension of this relationship is likely to facilitate the development of novel treatment strategies for bladder cancer.

### 3.9 The SWI/SNF Complex Regulates the R‐Loop

Transcription‐replication conflicts constitute a major spontaneous origin of genomic instability, which is frequently associated with R‐loop, a structure encompassing DNA–RNA hybrids and displaced single‐stranded DNA. Chromatin factors have emerged as significant participants in the stability of R loops and related genomic instability [73].

The loss of ARID1A in tumour cells triggers anti‐tumour immunity by activating the STING‐I type interferon pathway via the R‐loop‐derived cytoplasmic DNA. This determines the potential molecular mechanism underlying the anti‐tumour immunity and immunotherapeutic response observed clinically in ARID1A mutant cells. ARID1A deficiency induces the accumulation of R‐loops, generates cytoplasmic DNA, and subsequently activates the STING pathway, thereby driving the expression of ARID1A‐IFN features. This discovery reveals the molecular mechanism through which ARID1A mutations promote ICB responses and may possess transformative application value in improving the selection of ICB patients, providing theoretical support for enhancing ICB efficacy through the use of cBAF (Subtypes of the SWI/SNF complex) inhibitors [53]. Following DNA double‐strand breaks, the formation of R‐loop is requisite for ARID1A to effectively recruit to the double‐strand break sites within transcriptional genes. Once the ARID1A‐containing cBAF complex (Subtypes of the SWI/SNF complex) is facilitated to recruit to the DSB site through R‐loop formation, ARID1A facilitates the recruitment of Ribonuclease H1 (RNaseH1). In unperturbed cells deficient in SWI/SNF factors such as BRG1 (It is encoded by the SMARCA4) and ARID1A, R‐loop levels escalate, giving rise to transcription‐replication conflicts and augmented genomic instability; moreover, the study indicates that the ARID1A‐containing cBAF complex (subtypes of the SWI/SNF complex) promotes the recruitment of RNaseH1 and RAD52, concurrently facilitating the rapid disintegration of R‐loop, further regulating DNA repair [74]. The depletion of BRG1 gives rise to an increment in S‐phase R‐loop, inducing replication forks to stall and resulting in DNA breaks throughout the entire nucleus. BRG1 directly acts upon R‐loop gain genes to prevent R‐loop accumulation. BRG1 and Fanconi anaemia complementation group D2 (FANCD2) are mutually interdependent on R‐loop, and BRG1 collaborates with the Fanconi anaemia (FA) pathway to resolve transcription‐replication (T‐R) conflicts to prevent R‐loop accumulation and related DNA damage, thereby addressing genomic instability [73].

In bladder cancer, studies have emerged revealing a close connection between it and the formation of R‐loop. The transcription factor ZBTB11 suppresses the proliferation and colony‐forming ability of bladder cancer cells through knockdown of its expression. ZBTB11 activates the transcription of DDX1 by binding to its promoter region, and Knockdown of ZBTB11 increases R‐loop accumulation and DNA damage, thereby inhibiting the growth of bladder cancer cells [75], the R‐loop interacts with chromatin remodelling complexes to regulate the stability of the genome [73], in bladder cancer, multiple subunits of chromatin remodelling complexes undergo mutations and are closely associated with cell proliferation and tumour progression. This might imply that the SWI/SNF complex could also play a role in bladder cancer by regulating the formation of R‐loop; however, the specific mechanism still requires further research for clarification. Although there is no explicit research indicating that this mechanism functions in bladder cancer, it provides us with more ideas for researching bladder cancer therapies and the future directions we should pursue.

## 4 Treatment for Bladder Cancer Targeting the SWI/SNF Complex

Urothelial carcinomas account for over 95% of bladder tumours and can be further categorised into non‐muscle‐invasive bladder cancer (NMIBC) and MIBC [76]. For NMIBC, transurethral resection of bladder tumour (TURBT) serves both diagnostic and staging purposes while also representing a pivotal therapeutic intervention [2], which may be complemented by immunotherapy [77] or intravesical maintenance chemotherapy [78, 79]. Radical cystectomy stands as the standard approach for localised MIBC [80], with neoadjuvant cisplatin‐based chemotherapy demonstrating efficacy in enhancing patient survival rates [81], additionally, patients may opt for a triple therapy regimen based on individual circumstances [2]. Despite local treatments such as radical cystectomy, patients with MIBC still face substantial risks of metastasis and recurrence [2]. Cisplatin‐based combination chemotherapy emerged as the gold standard for metastatic bladder cancer in the early 1990s [2], however, numerous patients are ineligible for cisplatin‐based regimens [82]. Notwithstanding these diverse treatment modalities, many patients remain incurable at present [76]. None of these approaches involve specific genetic alterations in bladder cancer; nevertheless, further exploration and expansion of genetic‐ and molecular‐based methodologies will reveal a robust association between the progression of bladder cancer and SWI/SNF complex. (Table 2).

**TABLE 2:** Therapeutic potential of SWI/SNF complex‐specific targets in bladder cancer.

Therapy	Target	Medicine	Mechanism of Action	References
Immune checkpoint therapy	ARID1A mutation	Immune checkpoint inhibitors	In squamous bladder cancer, the finding of ARID1A mutation co‐existing with high PD‐L1 expression suggests that these patients may benefit from immune checkpoint inhibitor therapy.	[17]
	ARID1A mutation and high expression of CXCL13		When ARID1A gene mutation and high expression of CXCL13 are present in bladder cancer, the sensitivity to immune checkpoint therapy is increased.	[54]
Integrated therapeutic approach	Loss of ARID1A	Combination of PI3K inhibitors and EZH2 inhibitors	ARID1A‐deficient bladder cancer cells are sensitive to PI3K inhibitors, and combined use with EZH2 inhibitors can achieve synergistic anti‐tumour effects.	[60]
	Loss of SNF5	Cisplatin combined with EZH2 inhibitor	The inhibitor of EZH2, GSK12, can enhance the sensitivity of SNF5‐deficient cells to cisplatin.	[58]
Inhibit signalling pathways	Loss of SMARCB1	STAT3 inhibitors	The STAT3 inhibitor TTI‐101 can inhibit the growth and metastasis of SMARCB1‐deficient bladder cancer.	[23]
	Loss of ARID1A	PI3K inhibitors	Studies show that PIK3IP1 protein is upregulated in ARID1A‐deficient bladder cancer cells and inhibits the PI3K signalling pathway.	[60]
	Loss of SNF5	EGFR inhibitors	Gefitinib has shown increased sensitivity in bladder cancer cells with low SNF5 expression.	[58]
Targeted inhibition of EZH2 to regulate gene expression	Loss of SNF5	EZH2 inhibitors	SNF5 is antagonistic to EZH2, and EZH2 inhibitors may enhance the sensitivity of low‐expressing SNF5 bladder cancer cells to cisplatin.	[24, 58, 61]
	Loss of ARID1A	EZH2 inhibitors	Cells with ARID1A deletion in bladder cancer are more sensitive to EZH2 inhibitors.	[60]
Prognostic biomarkers	PBRM1		In bladder cancer cells, the mRNA and protein levels of PBRM1 are significantly lower than those in normal cells.	[36, 85]
	SMARCC1		Compared to patients with negative expression of SMARCC1, patients with high expression of SMARCC1 in bladder cancer have a lower survival rate.	[25]
	SMARCA2		In advanced muscle‐invasive bladder cancer samples, the expression of SMARCA2 protein was significantly lower than that of normal urothelium.	[24]
	ARID1B		Suppressing the expression of ARID1B can significantly reduce the proliferation, migration, and invasive ability of bladder cancer cells.	[22]
	SNF5		Compared to conventional urothelial carcinoma, tumours with complete loss of SMARCB1 exhibit lower mutation burden, single nucleotide variant, and copy number variant frequencies.	[83]
Small molecule drugs targeting protein subunits	SMARCA2/SMARCA4	ATPase inhibitors	In other tumours, studies have shown that when SMARCA2/SMARCA4 is inactive, the cells become more sensitive to DNA damage, making chemotherapy and radiation therapy more effective. Developing inhibitors for these proteins may enhance the therapeutic effects, and in bladder cancer, multiple subunits are inactive, which may also lead to similar therapeutic effects.	[24, 49, 87]
	ARID1A/ARID1B	ARID inhibitors	Cells with ARID1A defects are sensitive to DNA damage, and when ARID1B is also defective, this effect is amplified. Developing inhibitors that increase the DNA damage effects on cancer cells may be a new target for IR therapy. Since ARID1A is highly mutated in bladder cancer, this approach may also be effective for treating bladder cancer.	[17, 45]
	BRD7	Bromodomain Inhibitors	The loss of the bromodomain leads to cancer sensitivity to PARPi and CPT, while small molecules containing bromodomains, such as BRD7, occur mutations in cancer types such as bladder cancer, which may indicate that bromodomain inhibitors are effective for bladder cancer treatment.	[46, 92]

*Note:* The table above presents a summary of pertinent research on the SWI/SNF complex in bladder cancer, highlighting the therapeutic potential of specific targets within the SWI/SNF complex for treating bladder cancer.

### 4.1 SMARCB1

In individuals suffering from bladder cancer, the absence of SMARCB1 has been linked to the stimulation of the IL6/JAK/STAT3 signalling pathway. TTI‐101, a STAT3 inhibitor, has shown effectiveness in suppressing the proliferation and spread of bladder cancer with SMARCB1 (Encoding SNF5 protein) deficiency [23]. Furthermore, SNF5 (It is encoded by the SMARCB1) expression may influence the sensitivity of bladder cancer cells to chemotherapeutic drugs [58]. Notably, compared to conventional urothelial carcinoma, loss of SMARCB1 may indicate a more favourable prognosis for this subtype [83].

### 4.2 ARID1

In cases of squamous bladder cancer, the presence of ARID1A mutations alongside elevated levels of PD‐L1 expression suggests a potential advantage in utilising immune checkpoint inhibitor therapy [17]. Bladder cancer cells with ARID1A deletion exhibit increased sensitivity to EZH2 inhibitors and reliance on related signalling pathways, specifically the PI3K/AKT/mTOR pathway [60]. Simultaneous occurrence of mutations in the ARID1A gene and elevated expression of CXCL13 increases responsiveness to immune checkpoint therapy in patients. Therefore, identifying mutations in the ARID1A gene and levels of CXCL13 expression could assist in more accurate selection of urothelial cancer patients suitable for immunotherapy [54]. As a mutually exclusive subunit of ARID1A, ARID1B shares significant homology with its counterpart; moreover, an elevated level of ARID1B expression is associated with a negative impact on the overall survival prognosis of individuals diagnosed with bladder urothelial cancer [22].

### 4.3 PBRM1

PBRM1 (Encoding BAF180 protein) is located in the 3p21 region of chromosome 3, where structural variations affecting genes are prevalent among individuals with bladder cancer [84], this observation implies a pivotal role for PBRM1 as a cancer suppressor during bladder carcinogenesis. Notably, diminished mRNA and protein levels of PBRM1 are evident within bladder cancer cells relative to their normal counterparts; furthermore, reduced expression of PBRM1 correlates with decreased survival rates among patients afflicted by this malignancy [36, 85]. Although the precise molecular mechanisms governing gene regulation by the PBRM1 remain elusive within the context of bladder cancer pathogenesis at present time, it is unequivocal that it exerts repressive effects on genes while holding promise for clinical implications.

### 4.4 Other Subunits

Bladder cancer patients with elevated levels of SMARCC1 (Encoding BAF155 protein) expression demonstrate markedly decreased survival rates in comparison to those with low or absent SMARCC1 expression, strongly indicating the clinical significance of SMARCC1 as a novel diagnostic and prognostic marker for bladder cancer [25]. In high‐grade muscle‐invasive bladder cancer samples, the level of BRM (It is encoded by the SMARCA2) protein expression is significantly reduced when compared to that in normal urothelium, indicating a decrease in the levels of BRM expression [24]. The upregulation or downregulation of SWI/SNF complex subunits in bladder cancer may indicate the potential for assessing the status of bladder cancer and predicting related disease progression, suggesting a close association with disease progression.

## 5 Analysis and Discussion on Future Treatment Directions for Bladder Cancer

### 5.1 Development of Small Molecule Drugs Targeting Protein Subunits

The SWI/SNF complex plays a key role in chromatin remodelling and gene expression regulation, and its dysfunction is closely linked to the development of many types of cancer. Deeply exploring the development of drugs targeting the SWI/SNF complex has profound implications for cancer treatment and may open up new avenues for solving the cancer puzzle.

#### 5.1.1 Inhibitors Targeting the BRG1/BRM Protein

It is widely acknowledged that BRG1 (It is encoded by the SMARCA4) and BRM (It is encoded by the SMARCA2) are highly mutated subunits which are two ATPases capable of hydrolyzing ATP to generate energy [6]. The development of ATP enzyme inhibitors might prove more beneficial in the treatment of bladder cancer. The inactivation of BRG1 influences γH2AX phosphorylation [86], and interaction with acetylated γH2AX regulates repair [49], inactivation of BRG1 makes cells sensitive to DNA damage [26, 39, 49]. The inactivation or downregulation of BRM in BRG1‐mutated lung cancer cells would further render these cells IR‐sensitive [87]. Developing related inhibitors may enhance the effect of damaging cancer cells. In bladder cancer, diverse subunits of the SWI/SNF chromatin complex undergo dysregulation [24], furthermore, bladder cancer is closely related to DNA damage repair [47], the development of ATPase inhibitors might potentially result in superior treatment effects for bladder cancer. However, it is difficult to develop corresponding ATPase inhibitors for SMARCA2 (Encoding BRM protein) and SMARCA4 (Encoding BRG1 protein) due to their highly conserved bromodomain and ATPase domains [5]. A dual ATPase inhibitor (BRM014) developed by a certain company is capable of silencing the expression of the SMARCA2 target and demonstrating anti‐proliferative activity in cell lines with SMARCA4 mutations and xenograft models; however, these drugs exhibit poor tolerability in preclinical models [67]. Another company's small molecule (FHT‐1015) has also been reported to inhibit the ATP enzymatic activity of SMARCA2 and SMARCA4 via a mechanism distinct from that of BRM014 [5]. Developing small molecule enzyme inhibitors that bind only to one of the related homologous pairs has proven to be challenging, as their ATP enzyme domains are highly homologous (93% conserved). Subsequently, the development of PROTAC was employed to rapidly and completely degrade the target protein through the protein degradation mechanism. These bifunctional molecules comprise two linked ligands, one of which binds to the target of interest, and the other to the ubiquitin ligase protein degradation mechanism. Linking the target protein to the protein degradation mechanism will lead to the ubiquitination of the target protein and subsequent degradation by the proteasome [88]. The first PROTAC targeting SMARCA2 and SMARCA4 is a molecule called ACBI1, which utilises a bromodomain ligand to rapidly degrade SMARCA2, SMARCA4, and also PBRM1 (Encoding BAF180 Protein), another PBAF‐specific subunit, which has a similar bromodomain [89]. Later, a PROTAC was developed that uses a ligand that binds to the ATPase domain to only degrade SMARCA2 and SMARCA4, thus leaving PBRM1 intact [90]. However, neither of these degraders attained specificity for SMARCA2 in relation to SMARCA4, and there was no notable disparity in their selectivity for degrading SMARCA2 and SMARCA4. New generations of degraders, such as ACBI2, A947, and SMD‐3040, are endeavouring to ameliorate this situation and aspire to enhance the specificity of degradation for SMARCA2 [5], future development of BRM/BRG1 inhibitors demands further exploration and research.

#### 5.1.2 Inhibitors Targeting ARID (AT‐Rich Interaction Domain) Domain

ARID1A/B are mutually exclusive components of the cBAF complex (Subtypes of the SWI/SNF complex), and these ARID‐domain‐containing proteins are of particular interest because they can directly interact with DNA and are thought to be crucial for recruiting the SWI/SNF complex to different genomic sites [12]. ARID1B‐deficient cells show increased phosphorylation of CHK1, CHK2, ATM, and γH2AX, indicating strong DNA damage [44]. ARID1A affects the glucocorticoid pathway to regulate the cell cycle and DNA damage [43]. Cells lacking ARID1A are sensitive to PARPi both in vitro and in vivo, and the toxicity of PARPi can be enhanced when combined with low‐dose ionising radiation [42]. ARID1B is mutually exclusive with ARID1A and exists in the cBAF complex (One subtype of the SWI/SNF complex), and ARID1B knockdown selectively increases the radiation sensitivity of ARID1A‐mutated CRC cells [45]. The mutation rate of ARID1A in squamous bladder cancer is higher (15%) [17], so designing ARID1A inhibitors or PARPi may provide new treatment options for bladder cancer. Meanwhile, taking advantage of the interaction between ARID1B and PARPi, designing related inhibitors may enhance the therapeutic effect. ARID1A and ARID1B lack drug‐like properties that are easily accessible. High‐throughput screening identified BD98, a small molecule inhibitor targeting the SWI/SNF complex containing ARID1A [91]. The precise mechanism of action of this ligand is still unclear, however, preliminary data suggests that it binds to ARID1A and blocks complex assembly or interaction with chromatin. These findings indicate that ARID proteins are targetable, but ARID1B inhibitors may be more clinically relevant [5].

#### 5.1.3 Inhibitors Targeting Bromodomain‐Containing Proteins

Proteins possessing a bromodomain, such as BRD7 and PBRM1 (Encoding BAF180 protein), can bind to acetylated proteins through their bridging domain and are considered crucial for targeting the SWI/SNF complexes to specific genomic regions by interacting with acetylated histones [6]. The bromodomain subunit has been demonstrated to be significant for DSB repair. The inactivation of BRD7 elevates the sensitivity of cells to CPT and PARPi [46]. BRD7 mutations or downregulation are detected in numerous cancer types, including bladder cancer, endometrial cancer, hepatocellular carcinoma, and melanoma [92]. Given that cells lacking bromodomain subunits are more susceptible to DNA damage, the development of more potent and selective bridging domain inhibitors (BDi) is likely to enhance the efficacy of chemotherapy or radiotherapy in bladder cancer. Nevertheless, another bridging domain molecule, PBRM1, plays a distinct role in bladder cancer. It is mutated in 2%–10% of bladder cancers, and the low expression of PBRM1 is associated with a lower survival rate, PBRM1 seems to act as a tumour suppressor by suppressing the expression of cyclin B1 in bladder cancer [36]. Further exploration of PBRM1's role in bladder cancer is necessary, emphasising the need for designing individualised treatments for each molecule to achieve more precise and reliable therapeutic approaches. New compounds with specificity for BRD7 [93] and PBRM1 [94] have been developed, which might have the potential to selectively regulate pBAF (Subtypes of the SWI/SNF complex) activity.

### 5.2 Development of Combination Therapy

Given the heterogeneity of bladder cancer, the responses of patients to existing treatment regimens exhibit significant variations. Thus, exploring personalised treatment strategies based on specific molecular markers is indispensable for the treatment of bladder cancer. Further research is requisite to evaluate the efficacy and safety of these targeted therapies and to explore combination therapy strategies for enhancing therapeutic efficacy. For instance, ARID1A‐deficient bladder cancer cells are susceptible to PI3K inhibitors, and the combination of them with EZH2 inhibitors can generate synergistic antitumor effects. We can combine PI3K inhibitors with EZH2 inhibitors to augment the therapeutic effect [60]; SNF5 and EZH2 are mutually antagonistic, and EZH2 inhibitors may enhance the sensitivity of low‐expression SNF5 (It is encoded by the SMARCB1) bladder cancer cells to cisplatin. We can employ EZH2 inhibitors in combination with cisplatin [58]. BRD7 inactivation will increase the sensitivity of cells to camptothecin (CPT) and PARP inhibitors (PARPi) to a certain extent [46]. BRD7 mutations or downregulation occur in numerous cancers, including bladder cancer, endometrial cancer, hepatocellular carcinoma, and melanoma [92]. Given that cells lacking BRD7 subunits are more sensitive to DNA damage, the development of more efficacious and selective BDi may enhance the efficacy of chemotherapy or radiotherapy for bladder cancer. Combination therapy offers a more effective means for the treatment of bladder cancer; however, it also confronts numerous challenges. Further research is needed to optimise treatment regimens and improve the quality of life and survival rate of patients. Certain bladder cancer patients with mutations in key SWI/SNF complex subunits may be treated with small molecule inhibitors capable of regulating chromatin remodelling processes. This targeted therapy can target cancer cells more precisely and reduce damage to normal cells.

## 6 Identification of Biomarkers for SWI/SNF Mutations in Bladder Cancer

SWI/SNF complex mutations are prevalent in bladder cancer, and these mutations can impact the function of the complex and alter the biological behaviour of cells [24]. The identification of biomarkers associated with SWI/SNF mutations in bladder cancer is of significance for the diagnosis, treatment, and prognosis assessment of bladder cancer. These biomarkers can also be utilised to evaluate the survival prognosis of bladder cancer patients. For instance, the mRNA and protein levels of PBRM1 (Encoding BAF180 protein) in bladder cancer cells are conspicuously lower than those in normal cells, and the low expression of PBRM1 is associated with lower survival rates in bladder cancer patients [36]. The low expression of SMARCA2 (Encoding BRM protein) is associated with metastasis in MIBC patients and reduces their survival rate [24]. The absence of the ARID1A protein in bladder cancer is related to the tumour grade and stage [18]. These cases suggest that the prognosis of patients can be judged based on the expression of SWI/SNF subunits, and medical staff can provide more accurate prognosis information to patients based on these markers.

## 7 Conclusion

The SWI/SNF chromatin remodelling complex is crucial in biology as it regulates the connections between histones and DNA, leading to alterations in the arrangement and positioning of nucleosomes [28]. In the context of bladder cancer, dysregulation or reduced expression of the SWI/SNF complex and its specific subunits such as ARID1A [60] and SNF5 [58] is closely linked to tumour progression, offering a novel therapeutic target. Novel approaches to treating SWI/SNF complex mutations in bladder cancer in the future might involve the utilisation of immune checkpoint inhibitors [17, 54], signalling pathway inhibitors [23, 58, 61], EZH2 inhibitors [24, 58, 60, 61], among others. Despite numerous studies demonstrating the close association between bladder cancer and the SWI/SNF complex, there remain many unresolved questions. For instance, while some research indicates a functional antagonistic relationship between ARID1A and EZH2 [61], other studies show no correlation between ARID1A expression and EZH2 levels or H3K27 methylation in bladder cancer [18]. While some research indicates a potential link between ARID1A deletion and tumour advancement, other studies suggest that the expression of ARID1A shows a significant rise from normal tissue to non‐invasive urothelial carcinoma and subsequently to invasive urothelial carcinoma; additionally, reduced ARID1A expression appears to indicate a potential association with unfavourable outcomes for individuals diagnosed with bladder cancer [95].

The precise elucidation of the role of the SWI/SNF complex in bladder cancer necessitates an extensive exploration. Investigating the specific mechanisms underlying the function of these complexes in bladder cancer is imperative for devising more efficacious treatment modalities. Subsequent research should delve further into the regulatory impact of the SWI/SNF complex on gene expression and its influence on tumour initiation and progression. These endeavours may unveil novel therapeutic approaches that could ameliorate the prognosis of patients with bladder cancer.

## Author Contributions

**Zixiao Lei:** visualization (lead), writing – original draft (lead), writing – review and editing (lead). **Yanfeng Han:** investigation (supporting), writing – review and editing (supporting). **Jiejun Liao:** investigation (supporting), writing – review and editing (supporting). **Xiaohong Li:** investigation (supporting), writing – review and editing (supporting). **Qisheng Su:** conceptualization (lead), investigation (lead), project administration (lead), supervision (lead), writing – review and editing (supporting). **Zheng Yang:** conceptualization (lead), investigation (lead), project administration (lead), supervision (lead), writing – review and editing (supporting).

## Conflicts of Interest

The authors declare no conflicts of interest.

## Data Availability

The authors have nothing to report.
